# Further Validation and Test-Retest Reliability of the Spanish Version of the Standardised Assessment of Personality - Abbreviated Scale (SAPAS) for Personality Disorder Screening in Community Mental Health Settings

**DOI:** 10.62641/aep.v53i2.1895

**Published:** 2025-03-05

**Authors:** Andrés Arroyo-Sánchez, Renata Gómez Passalacqua, Jorge A. Cervilla, José Eduardo Muñoz-Negro

**Affiliations:** ^1^Departamento de Psiquiatría, Facultad de Medicina, Universidad de Granada, 18016 Granada, Spain; ^2^UGC Salud Mental, Hospital Universitario Clínico San Cecilio, Servicio Andaluz de Salud, 18016 Granada, Spain

**Keywords:** Personality Disorder, Standardised Assessment of Personality - Abbreviated Scale (SAPAS), International Personality Disorder Examination (IPDE), questionnaire validation, community mental health population

## Abstract

**Background:**

Personality Disorders (PDs) are a critical public health issue frequently misdiagnosed and underdiagnosed in mental health services. The purpose of this study is to demonstrate the reliability, validity and repeatability of the Spanish version of the Standardised Assessment of Personality - Abbreviated Scale (SAPAS), a short and self-administered scale for PD diagnosis and screening.

**Methods:**

This longitudinal study was performed using a 107-patient sample who attended community mental health services and outpatient clinics. A Receiver Operating Characteristic (ROC) curve was utilized to determine concurrent validity by comparing the SAPAS with the International Personality Disorder Examination (IPDE), thus establishing sensitivity, specificity, and predictive value for several cut-off points. Repeatability was measured by calculating an Intraclass Correlation Coefficient (ICC) between an initial SAPAS administration and a second one carried out 30 days later.

**Results:**

The Area Under the Curve (AUC) was found to be 0.84. A cut-off point of 3 provided 90% sensitivity and 52% specificity and correctly classified 71% of the cases. The ICC for the two SAPAS measures was 0.88.

**Conclusion:**

Our Spanish translation for the SAPAS proves to be a reliable, valid and consistent PD screening tool in mental health settings.

## Introduction

A Personality Disorder (PD) diagnosis is a frequent comorbidity found in patients with other psychiatric conditions [1, 2]. Furthermore, medical comorbidities are also often found in PD patients [3], who are also under risk for higher suicidality [4]. These patients show difficulties in social, recreational, occupational, and global functioning and are associated with an increased risk of neurotic or affective disorders [5]. One major risk factor for the development of PDs is having suffered stressful life events (SLEs) during childhood, including abuse, neglect, and other forms of early-age trauma [6]. In later adult life, PDs have been related to higher rates of unemployment and divorce and further forms of social and psychological adversity [7]. Several studies show that the prevalence of PDs ranges between 5.9% and 22.5% among the general population [8, 9, 10]. Meta-analyses and systematic review studies summarize such prevalence between about 7.8% [11] and 12.16% [12]. In Granada (Spain), a previous general population study estimated PD prevalence at 10.8% [13]. However, prevalence among mental health patients is much higher and imprecisely estimated to range from 40% to 92% in Europe [14]. Nonetheless, PDs are believed to be often underdiagnosed [15, 16] and/or misdiagnosed [17]. Furthermore, PD diagnosis is time-consuming and costly. Hence, there is evidence that the management and diagnosis of PDs represent an increase in the demand for health resources [18]. Until now, there has been a limited amount of PD screening tools in Spanish. One that has proven to be clinically reliable in several countries is the International Personality Disorder Examination (IPDE) [19], a relatively lengthy screening instrument with 59 items, for which a Spanish adaptation exists [20].

In this context, a shorter, simpler, valid and reliable self-administered screening scale in Spanish could prove clinically useful for the diagnosis of PD. A Spanish version of The Standardised Assessment of Personality - Abbreviated Scale (SAPAS) [21] could well be one such screening tool, since it has already shown excellent psychometric properties as a screening test for the English-speaking population [21]. The SAPAS is a self-administered screening scale consisting of 8 yes/no questions deriving from the Standardised Assessment of Personality (SAP) [22], a larger and lengthier scale. Amongst English-speaking samples, the SAPAS showed 94% sensitivity and 85% specificity in its first validation study [21]. Additional studies demonstrated its validity among patients with substance abuse [23], adolescent patients [24] and even the general population [25], although showing a lower predictive value of 58%. SAPAS adapted versions also exist in French [26], Japanese [27] and Bengali [28]. This instrument was initially designed using the reference of the 10th Edition of the International Classification of Diseases (ICD-10) PD classification, which was rooted in a categorical paradigm. However, this scale may still hold relevance within the new ICD-11 classification of PD [29] both from a categorical and dimensional viewpoint.

We previously published a preliminary validation study on a Spanish version of the SAPAS [30]. Even though this study achieved relevant figures for a first validation, indeed comparable with those of the original validation study in the English language (0.66 Internal Consistency Area Under the Curve (AUC) of 0.89; 84% sensitivity and 79% specificity at a 5 cut-off point), that study was limited in its sample size and by the fact that we did not report test-retest reliability. Therefore, the aim of this study is to consolidate the results achieved by our previous report, increasing sample size and testing for test-retest reliability (repeatability), which has not yet been done for this scale in our language. We hypothesise the SAPAS scale to be a useful tool for easily detecting potential PD cases, for the Spanish clinical population attending mental health services in outpatient clinics.

## Methods

### Study Design

A longitudinal study was performed to further test for reliability and validity of a double-translated (translated and back-translated) version of the SAPAS screening scale in Spanish. The study started in September 2019, but the sample taking stage had to be interrupted in March 2020 due to the Covid-19 pandemic. Thus, the study was resumed in September 2022 and was continued until May 2023.

### Sample Size

Assuming a 95% Confidence Interval (CI) and a PD prevalence of 37% in clinical populations in Granada [30], and considering a precision of 10% as appropriate due to the wide range of PD prevalence estimated in Europe throughout the literature (40–92%) [14], a sample size of 90 individuals was calculated to provide sufficient power for this study. However, we managed to increase the sample size to 107 individuals, which is estimated to provide more representative results, along with enough power for further testing validity and reliability, as previously suggested [30].

### Inclusion/Exclusion Criteria

Inclusion criteria were to accept any adult individual attending outpatient clinics at both, Clínico San Cecilio University Hospital and Virgen de las Nieves University Hospital (Granada, Spain), for recruitment provided they understood and could give informed consent to take part in the study.

Exclusion criteria were to reject individuals younger than 18 years and those who, due to the severe nature of their symptomatology and/or psychological disability, were unable to provide informed consent or those could not understand the process of the study and questions asked during the interview. This included patients suffering from severe manic/delusional states and patients with intellectual disability.

### Variables/Measuring Instruments

A. Sociodemographic variables of the individuals that met inclusion criteria (age, sex, educational level, marital and employment status).

B. Full psychiatric history, including previous diagnosis and medication, using clinical standards.

C. The SAPAS self-administered screening scale for PDs was administered twice: first at a first interview when entering the study and, secondly, again to test for repeatability 30 days later.

D. The IPDE questionnaire for ICD-10-based PD diagnosis [19] was also used. This includes an initial self-administered questionnaire of 59 yes/no items, followed by a semi-structured hetero-administered interview with 67 questions which can be scored from 0 to 2 points. The IPDE questionnaire renders Negative, Probable or Positive diagnosis for each ICD-10 PD diagnosis, using 3 or 4 as cut-off points depending on each specific PD being tested, and it also provides dimensional scores for each PD. The previously-translated Spanish version [20] was used.

### Procedure

In the first stage of the study, the Spanish SAPAS adaptation used by the previous validation study [30] (Appendix) was initially run by a psychiatrist during patient recruitment, who simultaneously collected information such as informed consent, sociodemographic information and psychiatric history. Subsequently, at a later moment, the diagnostic IPDE interview was also administered.

At the second stage of the study, a second SAPAS measure was performed by the psychiatrist 30 days later, enabling to check for test-retest stability. During this study, we enlarged the original sample of 59 patients from our previous report to a final sample of 107 participants (n = 107).

### Data Analysis

The statistical analysis was aimed at determining the SAPAS capacity to correctly identify patients with a positive ICD-10 PD diagnosis (as established using the IPDE questionnaire) and to identify the most optimal screening cut-off point for the SAPAS. To achieve this, Cronbach’s Alpha was first calculated to determine the internal consistency of the SAPAS, both overall and after omitting each item from the total score. This measurement of internal consistency aims to assess the reliability of the measurement and to determine whether the questionnaire includes only one or several constructs in its structure. It also shows how each of the scale items contributes to the scale’s overall reliability [31]. In addition, adequacy for factor analysis was checked by using the Kaiser-Meyer-Olkin Test (KMO Test) and Bartlett’s Test of Sphericity [32]. Furthermore, Varimax Rotation was used for Principal Component Analysis (PCA) to determine the factorial structure of the scale.

To identify criterion validity between the SAPAS screening scale and the IPDE semi-structured interview, a Receiver Operating Characteristic (ROC) analysis was then used, which considered “positive” IPDE PD diagnosis as the gold standard. Thus, SAPAS performance was assessed and the ideal cut-off score for predicting a diagnosis of any PD was identified by comparison with the IPDE interview. An estimate of the scale’s discriminatory performance was calculated by analysis of the AUC of the ROC curve obtained after using a sensitivity-specificity plot. Then, Pearson’s correlation was employed to test the association between dimensional scores of PD as identified by the IPDE and their equivalent factors emerging from the PCA performed on the SAPAS scores.

Finally, an Intraclass Correlation Coefficient (ICC, one-way random model, 95% CI) was calculated to check for test-retest stability between the two successive SAPAS measurements. All calculations were performed using the IBM SPSS Statistic 24 (IBM Corp, Armonk, NY, USA) [33], with a 0.05 level of significance.

## Results

### The Sample

Descriptive results of the 107 patients that took part in the study can be found in Table 1. The mean age was 39.5 years (Standard Deviation (SD) = 13.06). 40 individuals (37.4%) were found to be potential PD cases upon performance of the IPDE interview. Borderline Personality Disorder (BPD) was the most frequent PD diagnosis and was ascertained in 14 of those identified as having a PD (F60.32, 13.1%). Anankastic PD ranked second in frequency, with 11 potential cases (F60.5, 10.3%). It is worth mentioning that, out of the 40 identified by the IPDE as PD cases, 15 (14% of the sample) were found to have more than one PD diagnosis. Thus, 10 patients presented two concurrent PD diagnoses (9.3%), 2 of them presented three simultaneous diagnoses (1.9%), and 3 of them qualified for four PD diagnoses (2.8%).

**Table 1.** **Description of the sample (n = 107)**.

Gender		
	Female	58%
	Male	42%
Marital status		
	Single	51%
	Married/long term partner	40%
	Divorced	7%
	Widowed	2%
Employment status		
	Unemployed	32%
	Studying/learning	16%
	Housekeeping/relative caretaker	2%
	Under contract	28%
	Long-term sick leave	15%
	Retired	6%
	Other	1%
Academic level		
	Knows how to read/write	4%
	Primary education	30%
	Secondary education	22%
	University degree (higher education)	40%
	PhD	4%
Axis I diagnosed patients (n = 66)		
	Affective (depression)	42%
	Affective (bipolar)	11%
	Neurotic	22%
	Psychotic	12%
	Other	13%
Pharmacological treatment		
	Antidepressants	33%
	Mood stabilizers	4%
	Benzodiazepines	26%
	Antipsychotics	13%
	None	24%

Frequencies are expressed in percentages.

### Reliability

Table 2 shows internal consistency of the Spanish SAPAS and its items following Cronbach’s α calculations. For the new sample tested, alpha coefficient was 0.60. The 8th item, “Generally a Perfectionist”, was the one that correlated the least with the other elements and omitting it from the analyses raised the alpha coefficient score to 0.62.

**Table 2.** **Internal consistency for the Spanish SAPAS**.

SAPAS item	Alpha coefficient if the item is omitted
Difficulty making/keeping friends	0.55
Usually a loner	0.50
Trusting others	0.55
Normally loses temper easily	0.56
Normally impulsive	0.58
Normally a worrier	0.59
Depends on others a lot	0.58
Generally a perfectionist	0.62
Total alpha coefficient	0.60

SAPAS, Standardised Assessment of Personality - Abbreviated Scale.

### Construct Validity

After performing a KMO Test and Bartlett’s Test of Sphericity, we found that the sample was suitable for factor analysis. Thus, statistical significance was achieved in both tests (KMO test = 0.599; Bartlett’s Test of Sphericity = 104.625; *p* ≤ 0.0001) and therefore, we proceeded with PCA. The solution extracted from the latter explained 58.1% of the total variance identifying three factors within the SAPAS items. The first and strongest factor (eigenvalue = 2.21) included the three first items of the scale: “Difficulty in making/keeping friends”, “Usually a loner” and “Trusting others”. The second factor (eigenvalue = 1.35) was mainly composed of items 4 “Normally loses temper easily” and 5 “Normally impulsive”. The last factor (eigenvalue = 1.10) included items 6 “Normally a worrier” and 7 “Depends on others a lot”. Item 8 “Generally a perfectionist” did not prove enough factor loading to correlate with any factor (0.158) and was left separate from the grouping. The factorial structure of the scale is depicted in Table 3.

**Table 3.** **Factor analysis for the Spanish SAPAS (construct validity)**.

SAPAS Item	Factor 1	Factor 2	Factor 3
Difficulty making/keeping friends	0.76	–0.08	0.13
Usually a loner	0.80	0.16	0.13
Trusting others	0.66	0.23	–0.05
Normally loses temper easily	0.17	0.79	0.12
Normally impulsive	0.11	0.80	0.05
Normally a worrier	0.07	–0.03	0.79
Depends on others a lot	0.10	0.12	0.77
Generally a perfectionist	0.38	–0.39	0.16
Eigenvalues	2.21	1.35	1.10
% Variance	27.63	16.81	13.70
% Total model variance	58.13		

### Concurrent Validity and SAPAS Cut-off Points

**Fig. 1.**
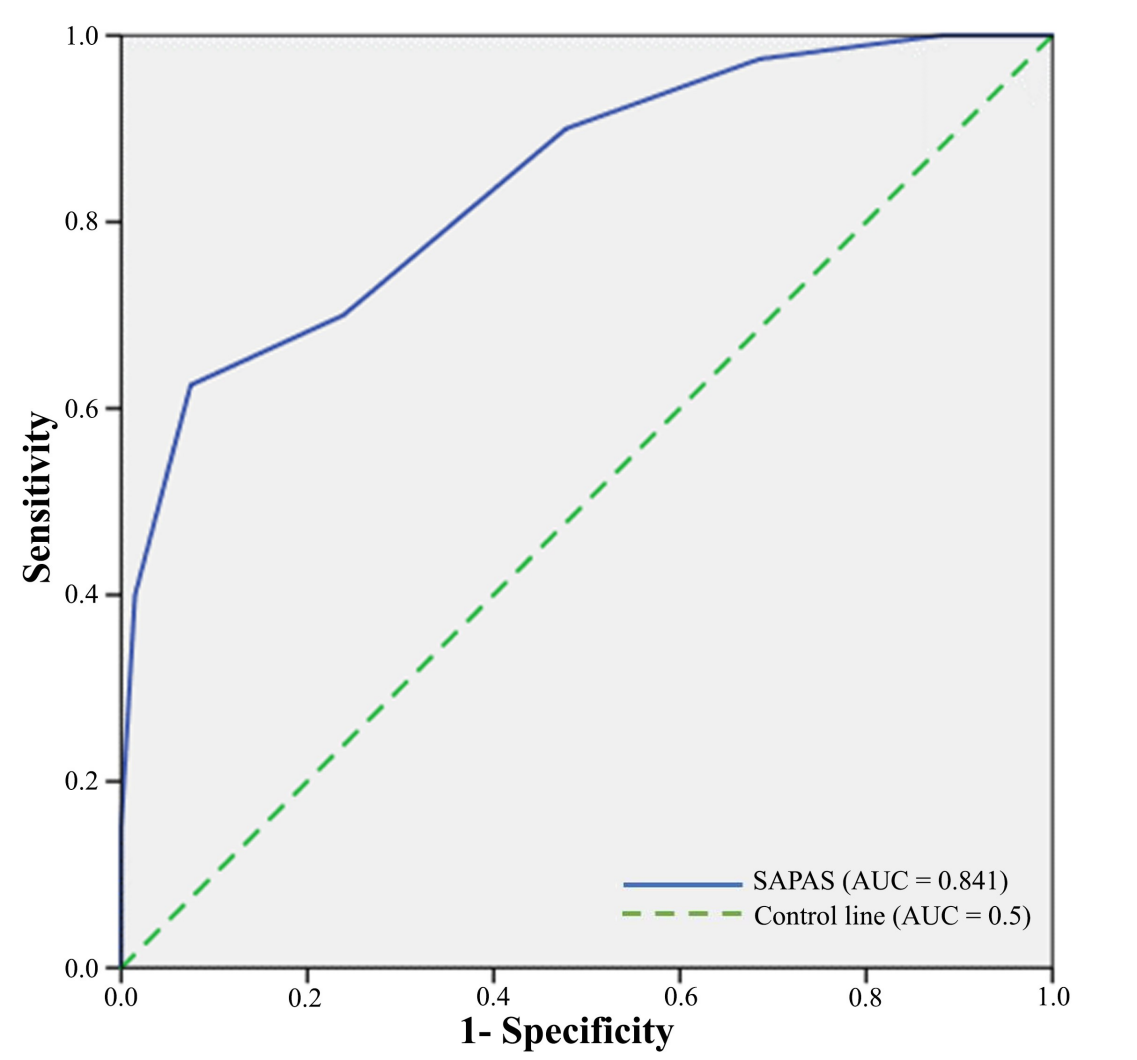
**ROC Curve for the SAPAS as a screening test for any IPDE ICD-10 PD diagnosis**. Area Under Curve (AUC) = 0.84 (95% CI, 0.76–0.92). ROC, Receiver Operating Characteristic; IPDE, International Personality Disorder Examination; ICD-10, 10th Edition of the International Classification of Diseases; PD, Personality Disorder.

As shown in Fig. 1, a ROC curve [34, 35] plotted the Spanish SAPAS scores as a screening test for PD diagnosis against the well-established IPDE-obtained specific diagnosis. Such ROC curve showed statistical significance (*p* ≤ 0.0001), with AUC amounting to 0.84 (95% CI = 0.76–0.92), suggesting that Spanish SAPAS could constitute a good screening tool, considering all possible cut-off scores (see Table 4).

**Table 4.** **Concurrent validity of the Spanish SAPAS: sensitivity, specificity, positive predictive value (PPV), negative predictive value (NPV) and cut-off points**.

Cut-off point	Sensitivity	Specificity	PPV	NPV	Correctly classified (%)
2 or more	0.96	0.31	0.07	0.93	63.5%
3 or more	0.90	0.52	0.18	0.82	71%
4 or more	0.70	0.76	0.33	0.67	73%
5 or more	0.63	0.93	0.21	0.79	78%
6 or more	0.40	0.99	0.31	0.69	69.5%
7 or more	0.15	1.00	0.91	0.09	57.5%

Table 4 shows the performance of the SAPAS at several cut-off points. A cut-off point of 4 allowed for a decent balance between sensitivity (70%), and specificity (76%), correctly classifying 73% of the individuals. However, the best performance for a screening tool was found at a cut-off point of 3, trading specificity (52%) for a significant increase in sensibility (90%), while still classifying 71% of individuals correctly.

When we explored the possible correlation between PD types and the three factors obtained by PCA on the SAPAS, we found that such factors broadly do correlate with the categorical splitting of PDs by clusters (A, B and C) (see Table 5).

**Table 5.** **Pearson’s correlation between dimensional scores of IPDE diagnoses and the factorial solution extracted for the Spanish SAPAS scores**.

IPDE Diagnoses		Corresponding SAPAS factor correlation
“Cluster A” Diagnoses		Factor 1 (Items 1–3)
	Paranoid PD	0.18 (*p * < 0.058)
	Schizoid PD	0.44 (*p * < 0.001)
“Cluster B” Diagnoses		Factor 2 (Items 4–5)
	Antisocial PD	0.32 (*p * < 0.001)
	Impulsive PD	0.66 (*p * < 0.001)
	Borderline PD	0.62 (*p * < 0.001)
	Histrionic PD	0.44 (*p * < 0.001)
“Cluster C” Diagnoses		Factor 3 (Items 6–7)
	Anankastic PD	0.38 (*p * < 0.001)
	Anxious-avoidant PD	0.33 (*p * < 0.001)
	Dependent PD	0.44 (*p * < 0.001)

Correlation is statistically significant if *p* < 0.05.

### Test-Retest Stability (Repeatability)

The ICC calculated for testing the test-retest stability [36, 37] of the Spanish SAPAS also exhibited statistically significant results. A 0.938 alpha coefficient was achieved when determining reliability of the measure, and an overall 0.88 correlation (95% CI = 0.795–0.931) was found for the single measures. When taking the average measures into consideration, we found a 0.94 intra-class correlation coefficient (95% CI = 0.886–0.964).

## Discussion

Following a previous report, in this study, we further demonstrate that the Spanish version of the SAPAS is a valid, consistent and reliable screening instrument for PDs for use in patients attending mental health services. Moreover, PCA determined the presence of 3 PD factors explaining most of the scores’ variability and that, interestingly, broadly correspond with the standardly established PD clusters and types [38, 39]. Thus, as suggested by the SAPAS validation studies [21, 25, 30], the first PD factor roughly colludes with the “*schizo-paranoid*” group of disorders, as items included in the factor are intended to measure both schizoid and paranoid PDs. Accordingly, the second factor correlates significantly with the “*Emotional Instability*” PD cluster represented by SAPAS items 4 and 5 measuring mostly impulsivity and anger. Finally, the third PCA extracted factor correlates well with SAPAS items (6 and 7) measuring the “*anxious-dependent*” PD types (Cluster C).

However, this categorical clustering distribution of the scale and its items can diverge from the more modern dimensional model of PD included in the newer ICD-11 classification [40, 41]. Whilst the SAPAS was not designed with the ICD-11 dimensional PD classification in mind, as it is indeed an older scale born under the context of a categorical paradigm of PD, the results of the factor analysis performed could also help to allocate this scale within the scope of the modern dimensional model, as each of the factors found could account for the latter’s different personality traits [29]. After all, items in the SAPAS are in a way a summary rephrasing of PD criteria and, indeed, a larger total SAPAS score could be in direct relation with increasing PD severity, as suggested by our previous validation study for the Spanish SAPAS [30]. This notion is certainly supported by findings of previous studies such as one by Ball *et al*. [29] and another reporting a recent translation of the SAPAS into the Japanese language [27].

Item 8 of the SAPAS questionnaire enquires about perfectionism, which could be related to Anankastic PD. However, this item was found to be the one that deviated the most from the rest of the internal consistency and reliability measures. Omitting this item would improve the alpha coefficient when measuring the scale’s internal consistency, and it was also the one item that exhibited more inconsistency when testing for construct validity via factor analysis. Even though it was indeed the third most significant item when determining Factor 3, and therefore could potentially be paired with items 6 and 7 when defining this third factor, its contribution to factor loading was noticeably less than significance of items 6 and 7. Besides, this is not found to be an anomaly, as these findings are consistent with other SAPAS validation studies previously cited. Indeed, the original article by Moran *et al*. [21] determined a global reliability of the scale with an alpha coefficient of 0.68, which increased to 0.70 when item 8 was omitted. The same was reported by a subsequent study, this time among the general population, [25], and by our previous study in the Spanish population [30].

Our factorial solution for the SAPAS can also be linked to ICD-11 dimensional personality dysfunction. Hence, our Factor 1, which includes items that explore difficulty making friends, loneliness (items 1 and 2) and (lack of) trusting others (item 3), could be closely related to the ICD-11 detachment domain. Similarly, factor 2, as it includes items related to easy loss of temper (item 4) and impulsivity (item 5), may be associated with both disinhibition and dissociality traits. And finally, factor 3, including items that account for anxiety (item 6) and dependence (item 7), could relate to the negative affectivity domain. In addition, item 8 of the scale (perfectionism) would undoubtedly collide with the ICD-11 domain of anankastia which, in turn, could explain why item 8 tends to isolate from all other SAPAS items here and in previous reports [21, 25, 30].

When compared to the gold standard validated questionnaire (IPDE), concurrent validity found in the SAPAS was most satisfactory. We identified an AUC comparable to that of a previous validation study in a Spanish population [30] and to the AUC reported by the first validation study in the English population [21]. Sensibility and specificity values were quite adequate as well, reaching higher specificity values than the previous Spanish validation study, even though sensitivity was lower at the 4 cut-off point. This is also consistent with other previously published studies, in which a more representative, larger sample shows a small increase in the β-Error and a decrease in the number of cases sorted correctly [25, 42]. Furthermore, even though the cut-off point at 4 offers a decent trade between sensibility and specificity, setting the cut-off point at 3 might offer better performance as a screening test, increasing sensibility to 90% at the expense of lowering specificity, thus properly classifying 71% of the cases. This is again consistent with the original English validation study [21], in which a 4 cut-off point is related to more parallel sensitivity and specificity values with specificity being the higher value, and a 3 cut-off point excelling at screening performance with 94% sensitivity. These results are also consistent with those achieved by the large-scale study performed in 2019 [43], which included a sample of more than 50,000 individuals measured with the SAPAS to determine the links between the SAPAS factorial structure and the 5th Edition of the Diagnostic and Statistical Manual of Mental Disorders (DSM-5) Alternative Diagnostic Model for Personality Disorders (AMPD). Similar to our report here, that study found that establishing a cut-off point at 3 reliably identified PD cases well in a clinical sample, while a cut-off point of 4 provided a balanced trade between sensitivity and specificity, making it more optimal for a general sample population. Furthermore, our factorial solution could also relate to the one achieved by the latter study, as it consists of a three-factor solution that can somewhat relate to the DSM-5 AMPD. Namely, our three factors could be related to the detachment, xxternalizing and negative affectivity DSM-5 AMPD domains, respectively.

Additionally, the finding that our three PD factors correlate to PD Clusters and dimensional scores of IPDE diagnoses, which is a key finding for the study as it replicates a similar report using the English version [39]. Correlation was highest for the emotionally unstable PDs, both impulsive and borderline types. This becomes particularly relevant when considering these disorders to be the most prevalent and severe among PDs [44, 45], suggesting that the SAPAS could potentially become a screening tool for clinicians dealing with such complex disorder. Moreover, the fact that correlation is still proven between the SAPAS and the IPDE dimensional score of PDs could lead to a further application in both clinical settings and population studies. Even though there are Spanish scales for specific PDs (Borderline [46] or Schizotypal [47]), personality dimensions [48] and personality traits [49], the SAPAS is still the shortest and most easily-administered scale for PD screening in different settings and, indeed, this scale is still being used for research and clinical purposes both, in Spanish [13] and in international settings in its original English version [50].

However, to our mind, this study’s greatest strengths are the results achieved when testing for test-retest stability (repeatability) which demonstrate an excellent degree of intra-operator consistency. This is consistent with previous literature, showing even a better performance, such as a validation study by Germans *et al*. 2008 [51], where the SAPAS was posed as a screening tool.

### Limitations and Future Projects

Even though subclinical and general populations are both settings where the SAPAS might perform at its best, further research is needed to truly test and validate this scale in the Spanish population outside of a clinical setting. As proven by the general population study performed for the English original version, the scale might have less discriminative power or different optimal cut-off points [25]. Regarding clinical populations, further testing including larger sample sizes may also prove useful in addressing the scale’s validity when other, less prevalent PD diagnoses, are involved, such as Histrionic or Antisocial, for which the SAPAS has not proven to capture variance properly [29]. Another caveat is the lax inclusion/exclusion criteria used in this study, and the authors acknowledge that the wide array of included patients may have been a confounding factor.

Besides, the authors are aware of an issue rooted in the very conception of the SAPAS as a measuring tool. Thus, while this scale may excel at measuring interpersonal aspects of PD, it is somewhat limited at exploring the self-related aspects of the personality disorder construct, and hence, it may not be as accurate as more modern scales at capturing the severity aspect of the PD diagnosis, which is a relevant part of the new ICD-11 formulation. A new scale validation study in Spanish, that could incorporate some of the SAPAS’ ethos whilst enhancing its capacity to identify severity and dimensional domains could become a ground-breaking project leading to a more accurate, modern and complete screening scale.

Finally, we must address the fact that determining concurrent and construct validity is always an arduous task regarding PDs research, due to their ill-defined constructual nature, unlike what might occur when studying other mental disorders that might be more deeply rooted in psychiatric and psychological research.

## Conclusion

The SAPAS still proves to be a reliable tool for the screening of PD in the Spanish mental health population, and its validity and consistency are here further established. Our findings demonstrate that the SAPAS is a solid screening-tool in settings in which the dimensional aspects of personality disorders are gaining strength over the previously established categorical paradigm.

## Availability of Data and Materials

The data that support the findings of this study are available from the corresponding author, Jorge A. Cervilla, upon reasonable request.

## Appendix

Spanish version of the Standardised Assessment of Personality - Abbreviated Scale (SAPAS) used in the study.

Por favor, dé la siguiente explicación antes de proceder con las preguntas:

“Me gustaría hacerle unas preguntas sobre sí mismo. Sus respuestas me ayudarán a comprender mejor como es usted normalmente. Si la forma en que usted ha sido en las últimas semanas o meses es diferente a cómo es usted normalmente, por favor remóntese a cómo era usted normalmente.”

Nota: Marque aquella opción que el entrevistado crea que se repite con más frecuencia y en la mayoría de las situaciones.

1. *En general*, ¿tiene usted dificultades para conseguir o mantener amistades? S/N

2. *Normalmente* ¿se describiría a sí mismo como una persona solitaria? S/N

3. *En general*, ¿confía usted en los demás? S/N

4. *Normalmente *¿se encoleriza usted con facilidad? S/N

5. ¿Es usted *normalmente* una persona impulsiva? (por ejemplo: ¿se apresura con la mayoría de las cosas sin pensar en las consecuencias?) S/N

6. *Normalmente* ¿se preocupa usted en exceso? S/N

7. *En general*, ¿depende usted mucho de los demás? S/N

8. *En general, *¿es usted perfeccionista? (Asegúrese de que se aplica a la mayoría de las tareas-no sólo a áreas aisladas de su vida) S/N
